# Supplementary material for: Comparison of the performances of survival analysis regression models for analysis of conception modes and risk of type-1 diabetes among 1985–2015 Swedish birth cohort
Source: PLoS One. 2021 Jun 25;16(6):e0253389. doi: 10.1371/journal.pone.0253389 (PMC8232413; doi:10.1371/journal.pone.0253389)
Supplement: Attachment — Submitted filename: response to reviewers Diabetics r2.docx [file pone.0253389.s002.docx]

The Editor

PLOS ONE

Attn: Yiqiang Zhan
Academic Editor
PLOS ONE

PONE-D-21-00472R1
Conception modes and risk of Type-1 diabetes among 1985-2015 Swedish birth cohort: How robust are the survival analysis regression models?

Dear Dr. Fagbamigbe,

Thank you for submitting your manuscript to PLOS ONE. After careful consideration, we feel that it has merit but does not fully meet PLOS ONE’s publication criteria as it currently stands. Therefore, we invite you to submit a revised version of the manuscript that addresses the points raised during the review process.


Please submit your revised manuscript by Apr 30 2021 11:59PM. If you will need more time than this to complete your revisions, please reply to this message or contact the journal office at plosone@plos.org. When you're ready to submit your revision, log on to https://www.editorialmanager.com/pone/ and select the 'Submissions Needing Revision' folder to locate your manuscript file.

Please include the following items when submitting your revised manuscript:

- A rebuttal letter that responds to each point raised by the academic editor and reviewer(s). You should upload this letter as a separate file labeled 'Response to Reviewers'.
- A marked-up copy of your manuscript that highlights changes made to the original version. You should upload this as a separate file labeled 'Revised Manuscript with Track Changes'.
- An unmarked version of your revised paper without tracked changes. You should upload this as a separate file labeled 'Manuscript'.

Thank you. We have provided and submitted the revisions accordingly
**Review Comments to the Author**

Reviewer #1: My general comment from the first round of reviews regarding the aim of the study still stands, but if the other reviewers and editors are happy then I have no further comments. The authors have adequately addressed my specific comments.

Thank you

Reviewer #2: (No Response)

Thank you

Reviewer #3: Thanks for the opportunity to review this re-submission.
I think the paper has improved in clarity and presentation from the previous submission, but my main concerns have still not been addressed.

Specifically, now that the authors clarified that "This study is a method paper with an application", I think the paper should focus more on the models comparison (which is still just barely discussed):

- The difference in interpretation between accelerated failure time and proportional hazards models is not discussed. Why use one against the other, if they are just a re-parametrisation of each other?

Thank you. We have improved on the model comparison, especially in the discussion part as we have already explained the differences between the models in the introduction

- The differences between the fitted model coefficients (in the application) is not presented, so I am not sure the reader can appreciate what happens when a not-flexible-enough parametric form is assumed. Performing a step-wise procedure with AIC/BIC does not assess robustness, to my eyes;

Than you. The fitted model coefficients have been provided

- Still, there is no much reason to use the flexible parametric models if hazard ratios are the measure of interest here - the Cox model would work just fine, without needing any functional form assumption;

Thank you. The study was to compare different models. As you rightly said the Cox compared favourably with the flexible parametric models, although the latter has lower model fit parameters

- I still don't think there is strong evidence against proportional hazards. The right-hand-side plot of figure 2 doesn't really show violations of the assumptions, as it is hard to identify non-proportional hazards on the survival scale. Furthermore, if the authors believe there are non-proportional hazards, how are they accommodating that into the analysis?

We agree, the study was to compare different models. The proportional models compared well with other models. We have reflected these in our results, discussions, findings and abstracts. Nonetheless, the flexible model, as detailed in literature, has capability of handling non-proportional hazards. As stated in the methodology, we focussed on the hazard scale of the models to ensure that the estimates from the FPSR and CPH models are comparable.

- It's still not possible to compare a partial likelihood model (the Cox model) with models that are fitted using full likelihood with AIC/BIC, that hasn't been corrected;

This has been corrected

- I still don't agree with the conclusion that "the methods of analysis may not be connected with the contradictory findings in earlier studies", we just don't know as we don't see the comparison between all the different models that are being studied here. Further to that, we don't know what the true effect is, so we cannot exclude that both the Cox and the Royston-Parmar models get it wrong, I believe?

We have changed the sentence to read “Hence the methods of analysis may not be disconnected with the contradictory findings in earlier studies”

In conclusion, I still think the paper needs major modifications if the goal is to study the robustness of different survival regression models. If this is a method paper, I think it should focus more on the methodological part rather than on the application (which I think it's still the case).

Thank you for the constructive comments. As you stated, this study is a method paper with an application. So we have discussed the models and the applications.
