# Supplementary material for: Comparison of the performances of survival analysis regression models for analysis of conception modes and risk of type-1 diabetes among 1985–2015 Swedish birth cohort
Source: PLoS One. 2021 Jun 25;16(6):e0253389. doi: 10.1371/journal.pone.0253389 (PMC8232413; doi:10.1371/journal.pone.0253389)
Supplement: Attachment — Submitted filename: response to reviewers Diabetics r3.docx [file pone.0253389.s003.docx]

The Editor

PLOS ONE

Attn: Yiqiang Zhan
Academic Editor
PLOS ONE

PONE-D-21-00472R2
Comparison of the performances of survival analysis regression models for analysis of conception modes and risk of Type-1 diabetes among 1985-2015 Swedish birth cohort
PLOS ONE

Dear Dr. Fagbamigbe,

Thank you for submitting your manuscript to PLOS ONE. After careful consideration, we feel that it has merit but does not fully meet PLOS ONE’s publication criteria as it currently stands. Therefore, we invite you to submit a revised version of the manuscript that addresses the points raised during the review process.


Please submit your revised manuscript by Jul 12 2021 11:59PM. If you will need more time than this to complete your revisions, please reply to this message or contact the journal office at plosone@plos.org. When you're ready to submit your revision, log on to https://www.editorialmanager.com/pone/ and select the 'Submissions Needing Revision' folder to locate your manuscript file.

Please include the following items when submitting your revised manuscript:

- A rebuttal letter that responds to each point raised by the academic editor and reviewer(s). You should upload this letter as a separate file labeled 'Response to Reviewers'.
- A marked-up copy of your manuscript that highlights changes made to the original version. You should upload this as a separate file labeled 'Revised Manuscript with Track Changes'.
- An unmarked version of your revised paper without tracked changes. You should upload this as a separate file labeled 'Manuscript'.

If you would like to make changes to your financial disclosure, please include your updated statement in your cover letter. Guidelines for resubmitting your figure files are available below the reviewer comments at the end of this letter.

If applicable, we recommend that you deposit your laboratory protocols in protocols.io to enhance the reproducibility of your results. Protocols.io assigns your protocol its own identifier (DOI) so that it can be cited independently in the future. For instructions see: <http://journals.plos.org/plosone/s/submission-guidelines#loc-laboratory-protocols>. Additionally, PLOS ONE offers an option for publishing peer-reviewed Lab Protocol articles, which describe protocols hosted on protocols.io. Read more information on sharing protocols at <https://plos.org/protocols?utm_medium=editorial-email&utm_source=authorletters&utm_campaign=protocols>.

We look forward to receiving your revised manuscript.

Kind regards,

Y Zhan
Academic Editor
PLOS ONE

Journal Requirements:

Please review your reference list to ensure that it is complete and correct. If you have cited papers that have been retracted, please include the rationale for doing so in the manuscript text, or remove these references and replace them with relevant current references. Any changes to the reference list should be mentioned in the rebuttal letter that accompanies your revised manuscript. If you need to cite a retracted article, indicate the article’s retracted status in the References list and also include a citation and full reference for the retraction notice.


Thank you. We have worked on this and ensured that all references are complete and correct. The old references 31 and 53 have been removed (there was no need for replacement) while old references 18, 32,35,36,28 t0 40 and 42 have been updated

6. Review Comments to the Author

Please use the space provided to explain your answers to the questions above. You may also include additional comments for the author, including concerns about dual publication, research ethics, or publication ethics. (Please upload your review as an attachment if it exceeds 20,000 characters)

Reviewer #3: Thank you for the opportunity to re-review this manuscript.
Overall, I think the focus of the manuscript has greatly improved: I have only a few (relatively) minor comments left to address, which are outlined below.

* "Model selection criteria" section, page 10, robustness of the model wasn't really assessed anywhere, so I would suggest removing that and focussing on fit of the models to the available data.

We have removed the issue about robustness and focussed on fit of the models to the available data.

* "Test of equality of incidence rates of type-1 diabetes" section, page 12, I would suggest reporting the rate difference per 1,000 person-years (or 100,000, as the authors prefer) to improve readability, as the currently reported rates have up to 6 significant digits.

Thank you. We agree with you. We have removed the column with up to 6 digits

* Again, if the PH assumption is violated, then all PH models yield (possibly) wrong results and non-proportional hazards need to be incorporated in the models. I still don't think Figure 2 shows evident violations of PH, but if the authors believe so, then the analysis (and all the fitted models in the manuscript) need to be updated to reflect this. This is the most important issue that the authors should focus on, in my opinion.

Thank you for pointing this out. We have now updated our text to indicate that PH assumptions were not violated

* "Comparison of the models" section, page 14, estimates for PH and AFT models are not directly comparable (as the authors state in the methods section, page 7-8). Therefore, comparing the magnitude of the fitted coefficients does not make sense - this needs to be corrected.

We agree totally. We have corrected this statement

* "Discussion" section, page 18, AIC and BIC for the Cox model are higher because it uses partial likelihood - therefore it's an unfair comparison. This has been fixed elsewhere in the manuscript, but not here; I think it needs to be adjusted here too.

Thank you. We have fixed the sentence

* Line 450, page 19, there's a typo - I think the author references there is Rutherford, not Rutherfold.

Thank you. We have corrected this

* "Strength and limitation" section, I think that (as mentioned before) robustness is not really studied here, so I would reword there.

We appreciate this concern. Yes, we have removed robustness across the manuscript and replaced with model fit

* "Conclusion" section, please mention that the models performed similarly in this specific setting, but there's no guarantee that this will be the case with other data sources or with other diseases. It's hard to generalise these findings to other settings. It would also be great if the authors could discuss non-poportional hazards: as above-mentioned, they first state that there are non-PH but then use PH (or AFT) models, this needs to be adjusted. A general discussion of non-PH as a strength of FPMs (where it's easy to incorporate this) is also welcome - otherwise, I see no reason to not use the Cox model if relative risk is the main measure of interest and non-PH are ignored.

Thank you for this suggestion. It has been incorporated appropriately in the discussion and conclusion sections

* Some additional comments: 1) Panel D of Figure 1 is hard to read (and I think the caption "the lines for 'father not diabetic'..." is cut off?), and 2) the names in the first column of figure 5 should be updated to be more descriptive (not just the variable name used in Stata).

 Thank you for the constructive comments. We have fixed this. Actually, the lines for father not diabetic and mother not diabetic overlapped.

Also, we have reproduced the forest plots to ensure easy reading.
